# Agro-Industrial Residues as a Unified Adsorbent Platform for the Removal of Rare Earth Elements, Transition Metals, and Organic Micropollutants from Water

**DOI:** 10.1007/s00128-026-04356-2

**Published:** 2026-11-11

**Authors:** Luis F. O. Silva, Tito J. Crissien

**Affiliations:** 1 Universidad de la Costa, Calle 58 #55-66, Barranquilla, Atlantico, 080002 Colombia; 2 IDITEK, Carrera 53 #53-105, Barranquilla, Colombia

**Keywords:** Agro-industrial waste, Biosorbent, Beta-cyclodextrin, Activated carbon, Water remediation, Circular economy

## Abstract

Agro-industrial residues are produced in large volumes and are frequently discarded, yet they can be converted into adsorbents that address pressing water contamination problems. This Technical Note advances a unifying valorization concept by re-analyzing the reported performance of four residue-derived adsorbents prepared from orange peel, potato peel, and rice husk. Two preparation routes are compared, namely the green citric acid grafting of beta-cyclodextrin onto fruit and tuber peels, and the purification or activation of carbonaceous rice husk co-products. Across the dataset, the same logic removed cationic species (lanthanum, iron, nickel), recalcitrant organics (phenol), and emerging contaminants (perfluorosulfonic acids), with reusability through mild ultrasound or thermal desorption and effectiveness in real matrices. Matching residue and modification route to contaminant charge and hydrophobicity emerges as a practical design rule. The analysis positions agro-industrial residues as a modular, low-cost adsorbent platform for environmental remediation.

## Introduction

The processing of food and crops leaves behind enormous quantities of organic residue. Orange peel alone is generated at roughly 32 million tons per year, and chemically modified peel has long been studied as a sorbent for pollutants (Feng et al. 2011). Rice husk is among the most abundant lignocellulosic by-products of agriculture and is repeatedly discarded as low-value waste even when it is rich in silica and carbon (dos Santos et al. 2024). Potato peel is a daily, zero-cost residue whose valorization is needed to avoid landfilling (Ebrahimian et al. 2022). Because adsorption is simple, inexpensive, and adaptable, converting these residues into adsorbents offers a route that treats one waste with another while supporting circular use of biomass (Crini et al. 2018).

The contaminants reaching water bodies span very different chemistries. Rare earth elements such as lanthanum are critical for modern technologies, yet their recovery from waste streams remains difficult (Hossain et al. 2022; Iftekhar et al. 2018). Transition metals, phenolic compounds, and per- and polyfluoroalkyl substances each demand distinct removal strategies, the last being especially persistent because of their stable carbon-fluorine bonds (Brase et al. 2021). Phenol is a priority pollutant in produced water and other industrial effluents (Allahkarami et al. 2022; Neff et al. 2011). This Technical Note advances a single design concept rather than reporting new experiments. By compiling and comparing four recent residue-derived adsorbents, it shows that one waste-to-adsorbent logic can be matched to cationic, recalcitrant organic, and emerging contaminants, and it proposes selection rules that link residue and modification route to contaminant charge and hydrophobicity.

## Materials and Methods

Performance data were compiled from four recently published studies by the present group, covering orange peel grafted with beta-cyclodextrin for lanthanum (Vieira et al. 2024), rice husk activated carbon for iron and nickel (dos Santos et al. 2024), ultrafine potato peel grafted with beta-cyclodextrin for perfluorosulfonic acids (da Silva Bruckmann et al. 2024), and an acid-base activated rice husk carbon for phenol (Reis et al. 2024). For each system the precursor, modification route, target contaminant, optimal pH, best-fit kinetic and equilibrium models, maximum adsorption capacity, thermodynamic nature, regeneration technique, reuse cycles, and performance in real matrices were extracted as reported in the source studies. No new primary measurements were performed; all numerical values reproduce those of the cited works. The comparison was organized around the relationship between the charge and hydrophobicity of each target and the surface chemistry of the adsorbent, in order to derive transferable design rules (Del Valle 2004; Kose et al. 2022).

## Results and Discussion

Two preparation routes underpin the four adsorbents (Table 1). In the first, beta-cyclodextrin is grafted onto fruit or tuber peel using citric acid as a green crosslinker and potassium dihydrogen phosphate as catalyst, forming ester bonds that introduce abundant carboxyl groups together with the hydrophobic beta-cyclodextrin cavity (Vieira et al. 2024; da Silva Bruckmann et al. 2024). The total carboxyl content reached 7.6 and 4.54 mmol per gram for the orange peel and potato peel materials, while the active beta-cyclodextrin content was 0.04 and 0.126 mmol per gram, reflecting different cavity accessibility (Del Valle 2004; Crini et al. 2018). In the second route, the sodium silicate-impregnated carbon that arises as a co-product of biogenic silica manufacture from rice husk is purified or activated, which removes silicate, opens the pore network, and raises the specific surface area to 753.0 and 1001.8 square meters per gram after washing and acid-base activation, respectively (dos Santos et al. 2024; Reis et al. 2024).

**Table 1:** Agro-industrial residue-derived adsorbents, preparation routes, target contaminants, and performance in synthetic and real aqueous matrices. Values are drawn from the indicated source studies

Residue	Adsorbent and modification	Target contaminant	Optimal pH	Maximum capacity	Real matrix removal	References
Orange peel	OP-beta-CD, citric acid grafting of beta-cyclodextrin	Lanthanum	5	95.79 mg/g (328 K)	Phosphogypsum leachate, > 80% at > = 5 g/L	Vieira et al. (2024)
Rice husk	AC-T, washed sodium silicate carbon co-product	Iron and nickel	3 (Fe), 6 (Ni)	Fe 1853.5; Ni 211.3 mg/g	Landfill leachate, Fe 81.5%, Ni 91.0%	dos Santos et al. (2024)
Potato peel	UFPPW-beta-CD, citric acid grafting of beta-cyclodextrin	PFSAs (PFHxS, PFPeS, PFBS)	3	PFHxS 100.9; PFBS 49.9 ug/g	Not tested (synthetic)	da Silva Bruckmann et al. (2024)
Rice husk	CAM, acid-base activated biogenic silica residue	Phenol	6	23.9 mg/g (298 K)	Produced water, 73% TOC	Reis et al. (2024)

The contaminant scope and the governing mechanisms are summarized in Table 2. Cationic targets bind chiefly through electrostatic attraction to deprotonated carboxyl groups, which explains why lanthanum uptake is favored near pH 5 and follows a Langmuir monolayer with an endothermic and spontaneous character, reaching 95.79 mg per gram (Vieira et al. 2024). Iron and nickel on the rice husk carbon also fit the Langmuir model with pseudo-second-order kinetics and endothermic behavior, with optimal removal at pH 3 for iron and pH 6 for nickel (dos Santos et al. 2024). The anionic and hydrophobic perfluorosulfonic acids instead rely on host-guest inclusion within the beta-cyclodextrin cavity, so that capacity increases with the length of the carbon-fluorine chain and the process is exothermic and physical, with the Sips model describing equilibrium (da Silva Bruckmann et al. 2024; Brase et al. 2021). Phenol removal on the high-surface-area carbon is likewise exothermic and follows the Sips and Liu models, consistent with pore filling (Reis et al. 2024; Allahkarami et al. 2022).

**Table 2:** Governing kinetic and equilibrium models, thermodynamic nature, dominant interactions, and regeneration routes for the four adsorbents. Values are drawn from the indicated source studies

Adsorbent	Kinetic model	Equilibrium isotherm	Thermodynamics	Dominant interaction	Desorption and reuse	Reference
OP-beta-CD	Pseudo-first-order	Langmuir	Endothermic, spontaneous	Electrostatic carboxyl-La binding, monolayer	Ultrasound with citric acid, 5 cycles	Vieira et al. (2024)
AC-T (rice husk)	Pseudo-second-order	Langmuir	Endothermic, spontaneous	Surface complexation and electrostatic	Basic leaching with NaOH, 5 cycles	dos Santos et al. (2024)
UFPPW-beta-CD	General order	Sips	Exothermic, spontaneous	Host-guest hydrophobic inclusion; H-bonding	Acetone with ultrasound, 7 cycles	da Silva Bruckmann et al. (2024)
CAM (rice husk)	Pseudo-second-order	Sips and Liu	Exothermic, spontaneous	Pore filling on high surface area carbon	Thermal at 180 °C, 15 cycles	Reis et al. (2024)

Reusability is a shared strength of both routes and depends on mild desorption (Table 2). The grafted polymers release the captured species under ultrasound, using dilute citric acid for lanthanum with about 95% recovery over five cycles, and acetone for the perfluorinated acids with more than 96% desorption in 25 min over seven cycles (Vieira et al. 2024; da Silva Bruckmann et al. 2024). The carbons are regenerated by basic leaching for iron and nickel across five cycles and by thermal desorption at 180 °C for phenol across fifteen cycles, indicating that the materials retain their structure under the chosen regeneration conditions (dos Santos et al. 2024; Reis et al. 2024).

Effectiveness in real matrices distinguishes these systems from many laboratory studies. The orange peel material removed more than 80% of lanthanum from a phosphogypsum leaching liquor that contained competing rare earth elements and a high calcium load, linking adsorbent design to the recovery of critical raw materials from industrial waste (Vieira et al. 2024; Lutke et al. 2022). The rice husk carbon treated a real landfill leachate, removing 81.5% of iron and 91.0% of nickel in a binary system and meeting reuse limits for irrigation (dos Santos et al. 2024). The activated carbon removed 73% of the total organic carbon from oilfield produced water, a complex saline matrix where phenol coexists with hydrocarbons and salts (Reis et al. 2024; Neff et al. 2011).

**Fig. 1:**
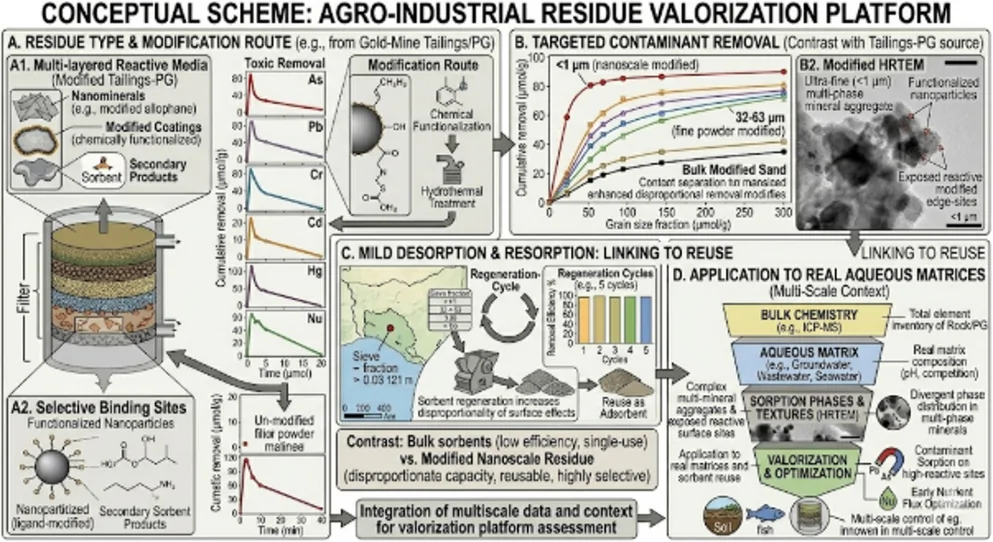
Conceptual scheme of the agro-industrial residue valorization platform, linking residue type and modification route to the targeted contaminant class, mild desorption for reuse, and application to real aqueous matrices

Taken together, the four cases support a single valorization concept in which the residue and its modification are matched to the contaminant. Carboxyl-rich surfaces suit cationic metals and rare earth elements, the hydrophobic beta-cyclodextrin cavity suits hydrophobic and anionic micropollutants such as perfluorinated acids, and high-surface-area carbons suit small neutral organics such as phenol (Vieira et al. 2024; da Silva Bruckmann et al. 2024; Reis et al. 2024). Figure 1 summarizes this platform from residue to real-matrix application. The main limitations to address before scale-up are standardized cost and energy accounting, competitive adsorption in multi-ion effluents, and fixed-bed operation, all of which would strengthen the case for deploying agro-industrial residues as a modular and low-cost remediation resource (Ebrahimian et al. 2022; Kose et al. 2022).

## Declarations

### Use of Artificial Intelligence

An artificial intelligence based language tool was used to assist with English copy editing, with the organization and formatting of references according to the journal style, with checking compliance against the journal guidelines. The authors reviewed and edited all text and take full responsibility for the content of this publication
